# The Antimicrobial Extract Derived from *Pseudomonas* sp. HP-1 for Inhibition of *Aspergillus flavus* Growth and Prolongation of Maize Seed Storage

**DOI:** 10.3390/foods14101774

**Published:** 2025-05-16

**Authors:** Marhaba Kader, Liping Xu, Longteng Fang, Reziyamu Wufuer, Minwei Zhang, Nan Wei, Dong Wang, Zhiwei Zhang

**Affiliations:** 1 School of Life Science and Technology, Xinjiang University, Urumqi 830017, China; 107552203666@stu.xju.edu.cn (M.K.); 107552303680@stu.xju.edu.cn (L.X.); 107552403438@stu.xju.edu.cn (L.F.); zhang78089680@sina.com (M.Z.); 2 School of Pharmaceutical Sciences, Institute of Materia Medica, Xinjiang University, Urumqi 830017, China; reziya04@xju.edu.cn; 3 Institute of Agro-Products Storage and Processing, Xinjiang Academy of Agricultural Science, Urumqi 830091, China; weinan9871@163.com

**Keywords:** *Pseudomonas*, antimicrobial extract, antifungal activity, maize seed, 1-phenazinecarboxylic acid

## Abstract

Maize, one of the most widely cultivated crops globally, is highly susceptible to mycotoxin contamination. In this study, an endophytic strain *Pseudomonas* sp. HP-1, isolated from *Peganum harmala* L., demonstrated significant biocontrol potential. The culture extract of *Pseudomonas* sp. HP-1 (PHE) exhibited strong antifungal activity, with inhibition zones of 40.07 ± 0.21 mm against *Penicillium italicum*, 29.71 ± 0.25 mm against *Aspergillus niger*, and 23.10 ± 0.44 mm against *A. flavus*, along with notable antibacterial activity against *Staphylococcus aureus* (22.43 ± 0.55 mm). At a concentration of 16 mg/mL, PHE almost completely inhibited the mycelial growth of *A. flavus*. The antifungal mechanism of PHE was investigated through scanning electron microscopy (SEM) and propidium iodide (PI) staining analysis, which demonstrated that antifungal activity is primarily through the disruption of cellular membrane integrity. Furthermore, PHE significantly reduced the incidence of *A. flavus* contamination in agroecological maize seeds during storage, and treated PHE showed superior antifungal efficacy compared to non-treated PHE, highlighting its potential as an effective antifungal agent for seed protection. Through one- and two-dimensional NMR and MS analyses, the primary active compound of PHE was identified as 1-phenazinecarboxylic acid. These findings indicate that PHE can be utilized as a sustainable antifungal agent for protecting maize seeds against mycotoxin-producing fungi.

## 1. Introduction

Maize (*Zea mays* L.), a globally vital food and feed crop, is a key source of dietary protein and calories [1]. In China, maize is the second largest grain crop, with an annual production of 260 million tons (2016–2020) and an area under cultivation of more than 40 million hectares [2]. However, post-harvest losses pose a significant challenge, with an estimated 2.4% annual loss attributed partly to fungal contamination during storage [3]. Fungal spoilage and mycotoxin contamination are pervasive issues in cereals and cereal-based products, with risks escalating throughout the supply chain, from cultivation to processing [4].

Fungal contamination of maize is caused by a diverse range of species, with over 20 reported, including *Aspergillus*, *Fusarium*, *Alternaria*, *Penicillium*, and *Stenocarpella* being the most prevalent [5]. These fungi not only deplete maize nutrients but also produce mycotoxins, posing serious health risks to humans and animals [6]. Among them, *A. flavus* is a major threat during maize storage, causing significant economic losses through aflatoxin production, which affects seed germination, metabolic processes, and nutritional value [7]. The infection rate of *A. flavus* can reach 10–20% during initial storage and escalate to 54–79% after six months [8]. Furthermore, fungal infection often initiates in the seed and root system, subsequently spreading to internodes, stems, and ears, leading to severe crop damage [9].

Traditional control methods rely heavily on chemical seed treatments, which raise concerns regarding environmental toxicity and human health risks [10]. Although new antifungal agents and synthetic preservatives have been introduced, their overuse has led to increased fungal resistance and environmental harm [11]. For instance, chemical fungicides like phytoconazole and phenyl ether metronidazole have been linked to adverse effects on ecosystems and non-target organisms [12]. The development of resistant fungal strains, ecological damage, and residual toxicity has further highlighted the limitations of synthetic chemicals [13]. In response to these challenges, there is growing interest in biological control methods as sustainable alternatives to synthetic fungicides [14]. Microbial biocontrol agents, particularly those producing antimicrobial metabolites, have gained attention for their potential in managing fungal infections [15]. For example, *bacillomycin* D from *Bacillus* sp. has demonstrated strong antifungal activity, reducing deoxynivalenol levels in stored maize [16]. Similarly, antifungal metabolites from *Streptomyces* sp. RL-1-178 have shown efficacy in inhibiting mycotoxin production during maize storage [17].

*Peganum harmala* is a perennial herbaceous plant native to Central Asia, North Africa, and the Middle East. Its primary bioactive compounds, β-carboline alkaloids, exhibit potent broad-spectrum antimicrobial activity against diverse bacterial and fungal pathogens [18]. Notably, *Pseudomonas* species dominate the symbiotic microbiome of *P. harmala*, many of which display antagonistic effects against pathogenic bacteria [19]. This antimicrobial activity may be attributed to secondary metabolites produced by *Pseudomonas*, such as pseudotrienic acids A and B (isolated from *Pseudomonas* sp. MF381-IODS), which inhibit *S. aureus* and *P. syringae* (MIC = 70 µg/mL for both) [20]. Furthermore, *Pseudomonas* sp., known for their role in plant disease control, produce a wide array of bioactive secondary metabolites, including phenazines, phloroglucinols, pyoluteorins, pyrrolnitrins, cyclic lipopeptides, hydrogen cyanide, lytic enzymes, and volatile organic compounds [21,22]. Despite their potential, limited research has explored the application of *Pseudomonas* sp. metabolites in maize grain storage. This study aims to (1) investigate the antimicrobial activity of metabolites from *Pseudomonas* sp. HP-1; (2) evaluate the efficacy of *Pseudomonas* sp. HP-1 extracts in preserving maize grain infected with *A. flavus*; and (3) identify the major antifungal compounds produced by *Pseudomonas* sp. HP-1.

## 2. Materials and Methods

### 2.1. Chemicals and Instrument

All chemicals and reagents used in this study were of analytical grade. Methanol, ethanol, dichloromethane, acetonitrile, Dimethyl sulfoxide (DMSO), and 1-butanol were obtained from Shanghai Titan Scientific Co., Ltd. (Shanghai, China). Culture media, including Luria-Bertani medium (LB), Potato Dextrose Agar medium (PDA), Mueller-Hinton Agar medium (MHA), and Mueller-Hinton Broth medium (MHB) were obtained from Qingdao Rishui Biotechnology Co., Ltd. (Qingdao, China). The following instruments were utilized in this study: a Nuclear Magnetic Resonance spectrometer (NMR, AVANCE NEO 600, Bruker, Germany), a High-Performance Liquid Chromatography system (HPLC, Agilent 1260, Agilent Technologies, Waldbronn, Germany), and a High-Resolution Mass Spectrometer (HR-MS, Ultimate 3000/Q-Exactive, Thermo Fisher Scientific, Waltham, MA, USA).

### 2.2. Isolation of the Strain HP-1

The strain HP-1 was isolated from the rhizosphere soil of *Peganum harmala* L. collected from Shirengou Village, Shuimogou District, Urumqi, Xinjiang, China (latitude 43°49′9″ N, longitude 87°46′27″ E). Soil samples were serially diluted under aseptic conditions, and the resulting suspension was spread onto Luria–Bertani (LB) agar plates. The plates were incubated at 25 °C for 14 days, after which a single colony was isolated and cultured on fresh LB agar medium to obtain a pure culture of strain HP-1. The strain HP-1 was identified through 16S rRNA gene sequencing. Universal primers 27F (5′-AGAGTTTGATCMTGGCTCAG-3′) and 1492R (5′-TACGGYTACCTTGTTACGACTT-3′). PCR products were sequenced by General Biosystems (Anhui), and the resulting sequences were analyzed using the NCBI BLAST algorithm (BLAST+, v2.16.0+) for similarity assessment. The 16S rRNA gene sequences have been deposited in the NCBI database with corresponding accession numbers.

### 2.3. Preparation of Culture Extract (PHE)

The strain HP-1 was cultured on LB agar medium at 30 °C for 4 days to ensure active growth. Subsequently, the strain was inoculated into a 500 mL conical flask containing 100 mL of SM seed medium (composed of soluble starch 1.0%, glucose 0.5%, tryptone 0.5%, yeast extract 0.2%, CaCO_3_ 0.3%, MgSO_4_·7H_2_O 0.05%, and K_2_HPO_4_ 0.1% in distilled water). The flask was incubated in a thermostatic shaker at 30 °C and 180 rpm for 4 days. Following seed culture preparation, 5 mL of the seed culture was transferred into fifteen 1000 mL conical flasks, each containing 200 mL of XM-3 production medium (composed of soluble starch 2.0%, glucose 0.5%, glycerol 2.0%, yeast extract 0.3%, polypeptone 1.5%, and HP20 1.0% in distilled water). The inoculated flasks were incubated on a shaker at 30 °C and 180 rpm for 7 days. After the fermentation period, 200 mL of 1-butanol was added to each flask, and the mixture was stirred for 1 h. The resulting mixture was centrifuged at 6000 rpm for 10 min to separate the organic layer from the aqueous layer containing mycelium. The organic solvent was evaporated under reduced pressure, yielding 4.2 g of crude extract from 3 L of culture broth.

### 2.4. HPLC-DAD Analysis of Culture Extracts

Analysis of the main chemical constituents of PHE by HPLC-DAD with minor modification according to the method of Yasmo Perez et al. [23]. The HPLC analysis was performed using a ZORBAX EC-C18 column (Agilent, Santa Clara, CA, USA, 4.6 × 150 mm, 4 μm) maintained at 32 °C. Detection was carried out using a diode array detector (DAD, Agilent, Waldbronn, Germany). The mobile phase consisted of 0.1% (*v*/*v*) formic acid in distilled water (solvent A) and acetonitrile (solvent B). The flow rate was set at 1 mL/min, and the injection volume was 100 μL. The total run time was 32 min, with gradient elution programmed as follows: 0–3 min, 5% B; 3–24 min, 5–85% B; 24–29 min, 85% B; and 29–32 min, 85–5% B.

### 2.5. Antimicrobial Effect of PHE

#### 2.5.1. Antibacterial Activity

The antibacterial activity against bacteria was determined using the Kirby–Bauer method [24] against two model strains, *E. coli* and *S. aureus*. Further, the stock cultures of bacteria were grown in the MHB medium at 37 °C for 24 h. The turbidity of the grown culture was adjusted with sterile water to 0.5 McFarland standard and diluted to 1 × 10^6^ CFU/mL of equivalent bacterial cells with sterile water. An aliquot (100 μL) of microbial suspension was spread on the surface of MHA media. The PHE was dissolved in DMSO to achieve a concentration of 2 mg/mL, and filter paper discs of 6 mm diameter were loaded with the PHE extracts and kept on MHA media plates. To evaluate the antibacterial potential of the PHE, kanamycin and dimethyl sulfoxide (1 mg/mL) were used as positive and negative controls, respectively. Finally, the media plates were placed in an incubator at 30 °C for 24 h, and the zones of inhibition were recorded in millimeters.

#### 2.5.2. Antifungal Activity

To evaluate the antifungal activity of PHE against yeasts, *C. albicans* and three predominant fungal cultures, *A. flavus*, *A. niger*, and *P. italicum* were used, wherein *C. albicans* used the Kirby–Bauer method, as described above. The agar well diffusion method was used to detect antifungal activity [25]. Spore counts of 1 × 10^6^ spores/m were obtained according to standard procedures for hemocytometer calculation. Fungal suspensions were placed in flasks containing 30 mL of sterile PDA at 50 °C with a spore count of 1 × 10^6^ spore/mL, and 6 mm diameter wells were punched out from the agar plate. The 100 μL of diluted PHE (10 mg/mL, dissolved in DMSO) was dispensed into each agar well in an MHA plate. The 100 μL of DMSO and Ampicillin B (1 mg/mL) served as negative and positive controls. The antibacterial activity was evaluated according to the inhibitory zone diameter after 24 h at 30 °C incubation (DMSO inhibitory effect subtracted).

#### 2.5.3. Inhibition of the Mycelial Growth Rate of *A. flavus*

The method proposed by Bluma and Etcheverry, with slight modification, was used to assess the effect of crude extracts on the growth rate of *A. flavus* filaments [26]. Briefly, PHE (dissolved in DMSO) was added to PDA (50 °C) to obtain solid culture medium containing 2 mg/mL, 4 mg/mL, 8 mg/mL, 12 mg/mL, and 16 mg/mL of PHE, respectively. After solidification, the agar block of *A. flavus* (6 mm) was placed in the center of the PDA plate containing the PHE. Control plates were PDA mixed with DMSO (1 mL) and the center was similarly inoculated with an agar block of *A. flavus*. Three groups each of treatment and control were incubated at 30 °C for 7 days and the whole experiment was repeated three times. The inhibitory rate was calculated according to the following formula:

(1) $$inhibitory\;rate=\frac{Rc-Rt}{Rc-6\;mm}\times 100\%$$

Rc is the average diameter of the control and the Rt is the average diameter of the treatment colony, respectively.

#### 2.5.4. Observation of Mycelium Morphology

Morphological changes in mycelium in the treatment groups were observed by scanning electron microscopy (SEM). *A. flavus* mycelia were obtained by culturing *A. flavus* spores (10^6^/mL) in 10 mL ×3 of SM medium (soluble starch 1.0%, glucose 0.5%, tryptone 0.5%, yeast extract 0.2%, CaCO_3_ 0.3%, MgSO_4_·7H_2_O 0.05%, K_2_HPO_4_ 0.1% added to distilled water) at 30 °C for 48 h. Fungal suspensions were treated with PHE (0, 8, 12, and 16 mg/mL) at 37 °C for 48 h. Briefly, mycelia were fixed with glutaraldehyde in phosphate-buffered saline (PBS) at 4 °C for 24 h, and dehydrated in ethanol for 15 min at each different concentration. Finally, the samples were freeze-dried, sprayed with gold, and observed through SEM.

#### 2.5.5. Determination of Cell Membrane Damage

The effect of PHE on cell wall integrity was measured using the method of propidium iodide (PI) staining [27]. In brief, *A. flavus* was incubated for seven days prior to treatment as outlined in Section 2.5.3. The untreated mycelia were used as a control group. The untreated mycelia were used as a control group. Mycelia were dyed with 25 μg/mL propidium iodide (PI) in the dark for 30 min at 37 °C and then washed three times and resuspended in PBS. Subsequently, fluorescence was detected by fluorescence microscopy (Leica DM3000-LED, Wetzlar, Germany).

### 2.6. Biocontrol Potential of PHE Against A. flavus on Maize Seeds

The maize samples were obtained from a local supermarket and sieved to remove impurities and randomly divided into four portions, each of 10 capsules. Before testing, the maize seeds were surface-sterilized by immersing them in a 0.05% sodium hypochlorite solution for 1 min, followed by several rinses with sterile distilled water. After drying the maize seeds in a sterile environment, each maize seed was infiltrated in the *A. flavus* solution of *A. flavus* (1 × 10^6^ spore/mL) for 10 min to ensure that each seed was completely contaminated. Each group of maize seeds was then treated with the solutions of PHE (100, 200, 300 mg/mL) and mixed for 5 min. Each set of maize seeds was laid flat in Petri dishes with PDA medium and incubated at 30 °C for 7 days for observation. DMSO was used as control and each treatment consisted of three replicates.

### 2.7. Isolation and Purification of Antibacterial Compounds

The crude extract PHE (3.0 g) was subjected to silica gel column chromatography with a step gradient of CH_2_Cl_2_/MeOH (1:0, 20:1, 10:1, 4:1, 2:1, 1:1, and 0:1 *v*/*v*). Fraction 2 (20:1) was concentrated to provide 85.5 mg of dried material containing the target compounds. The final purification was achieved by preparative HPLC (Agilent, Santa Clara, CA, USA, ZORBAX SB-C18, 9.4 × 250 mm, 5 μm), 4 mL/min, UV detection at 254 nm, eluted with a mixture of MeCN and 0.1% HCO_2_H solution (55:45) to yield HP-1 A (27 mg, *t*_R_ 8.0 min).

### 2.8. Statistical Analysis

Each test was conducted three times, and the mean ± standard deviation (X ± SD) was used to calculate the results. All data were analyzed by one-way analysis of variance (ANOVA) to determine the significance by IBM SPSS (Version 26.00) and plotted by Origin 2022.

## 3. Results and Discussion

### 3.1. Screening and Identification of Strain Pseudomonas *sp*. HP-1

Six soil samples were gathered from the rhizosphere soil of *Peganum harmala* L. All strains were isolated from soil samples by dilution spread plates on LB agar. These strains were cultured and fermented to obtain butanol extracts of the culture broths, which were analyzed by HPLC-DAD. It is noteworthy that the HPLC data for strain HP-1 had a high absorption peak at retention time 13.6 min, which showed characteristic absorbance at 200, 250, and 372 nm (Figure S1). Preliminary screening was conducted for the antimicrobial activity of PHE, which revealed significant antimicrobial activity. Consequently, strain HP-1 was selected as the target of this study. Moreover, the isolated strain HP-1 was identified as a member of the genus *Pseudomonas* on the basis of 98.4% similarity in the 16S rDNA gene sequence (873 nucleotides; DDBJ accession number PQ803664) to *Pseudomonas fluorescens* Dk3 (accession number KT377434). Plant symbiotic bacteria play a crucial role in protecting host plants from pathogenic microorganisms [28]. Some symbiotic bacteria are capable of producing antimicrobial metabolites, such as antibiotics, enzymes, or volatile organic compounds, which directly inhibit or kill pathogenic bacteria [29].

### 3.2. Antimicrobial Evaluation of PHE Against Microorganisms

**Figure 1:**
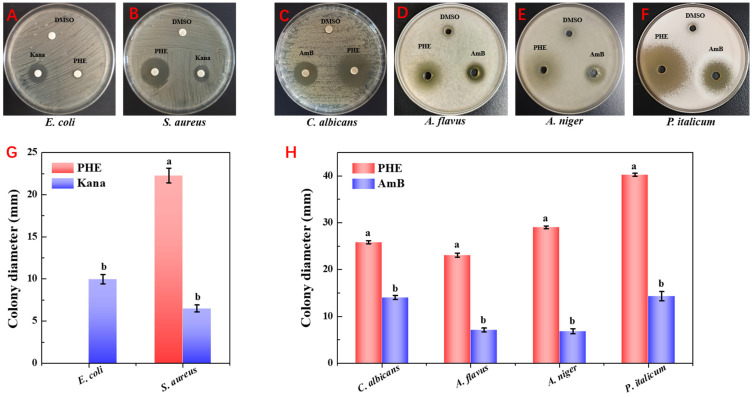
Inhibitory zone formed around the PHE against the six model strains. (**A**) PHE against *E. coli*. (**B**) PHE against *S. aureus*. (**C**) PHE against *C. albicans*. (**D**) PHE against *A. flavus*. and (**E**) PHE against *A. niger*. (**F**) PHE against *P. italicum*. (**G**) Comparison of antimicrobial activity of PHE against *E. coli* and *S. aureus* significantly, (a,b) non-identical letters denote statistical difference (*p* < 0.05). (**H**) Comparison of antimicrobial activity of PHE against *C. albicans*, *A. flavus*, *A. niger*, and *P. italicum* significantly, (a,b) non-identical letters denote statistical difference (*p* < 0.05).

The antimicrobial activity of the extracts was evaluated against six model strains using the agar well diffusion assay, including three fungi (*A. flavus*, *A. niger*, and *P. italicum*), two bacteria (*E. coli* and *S. aureus*), and one yeast (*C. albicans*). As illustrated in Figure 1, the PHE exhibited strong antifungal activity, with inhibition zones of 23.1 ± 0.44 mm, 29.71 ± 0.25 mm, and 40.07 ± 0.21 mm against *A. flavus*, *A. niger*, and *P. italicum*, respectively. Additionally, PHE showed notable antibacterial activity against *S. aureus* (22.43 ± 0.55 mm) but showed minimal activity against *E. coli.* The strong antifungal activity of PHE, particularly against *P. italicum* and *A. flavus*, highlights its potential as a sustainable biocontrol agent for protecting crops like maize from mycotoxin-producing fungi. Microorganisms synthesize antimicrobial compounds as secondary metabolites, such as PHE, which confer competitive advantages by inhibiting or eliminating competing microbial species. These bioactive substances exert their effects through multiple mechanisms, including inhibition of cell wall biosynthesis and disruption of cell membrane integrity. *Pseudomonas* sp. has been widely used as a model organism for biocontrol of plant diseases [30]. Such as *Pseudomonas syringae* strains ESC-10 and ESC-11 were successfully used to prevent post-harvest fungal diseases of citrus, pears, drupes, and potatoes during storage [31]. The ability of *Pseudomonas* to produce a range of antimicrobial agents (e.g., phenazines, phloroglucinols, pyrrolnitrins, and cyclic lipopeptides) that can antagonize fungal plant pathogens may be one of the main reasons [32].

### 3.3. Growth Inhibition of PHE Against A. flavus

**Figure 2:**
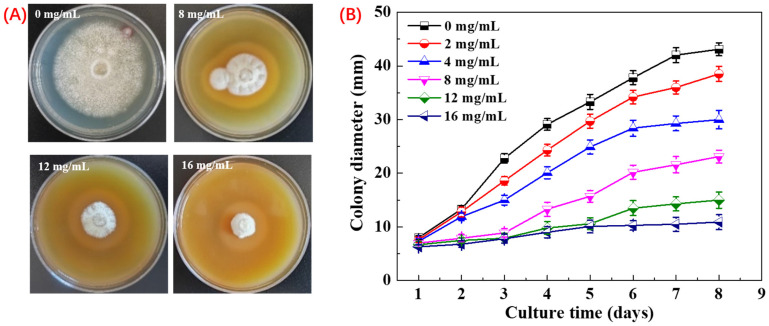
Inhibition of *A. flavus* by PHE at different concentrations. (**A**) Effects of different concentration of PHE on colony morphology at day 8. Values at different concentration differ significantly (*p* < 0.05), and (**B**) effects of different concentration of PHE on colony diameter from day 1 to day 8.

Inhibitory activity of PHE against *A. flavus* was assessed using the mycelial growth rate. The results demonstrated that PHE exhibited a strong inhibitory effect on *A. flavus* mycelial growth in a dose-dependent manner. After 8 days, significant differences were observed among the different concentrations of PHE. At concentrations above 4 mg/mL, the growth of *A. flavus* mycelia began to be inhibited. At 16 mg/mL, PHE displayed the most potent inhibitory activity, achieving a 90.3% inhibition rate (Figure 2). Microbial metabolites serve as the principal source of antibiotics, with many microorganisms producing bioactive secondary metabolites during growth that exhibit potent antimicrobial activity against competing microbial species [33]. Sawai investigated that the culture filtrate of *Streptomyces philanthi* RL-1-178 showed increasing inhibition of *A. flavus* with increasing concentration. After further research, one of the main compounds was found to be natamycin [17]. The PHE, as an extract of *Pseudomonas* sp. HP-1 fermentation broth, showed potential antigens *A. flavus* activity, and the key factor may be the production of antifungal secondary metabolites. Li et al. have reported that *Pseudomonas* produced the secondary metabolite phenazine-1-carboxamide, which has excellent activity against the fungus *Fusarium graminearum* [34].

### 3.4. Effect of PHE on Cell Morphology of A. flavus

**Figure 3:**
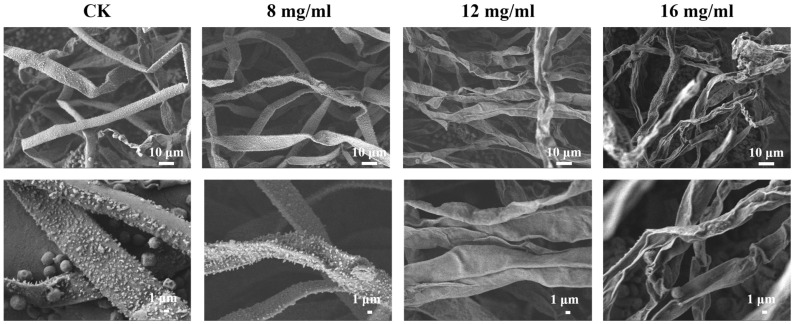
SEM images of *A. flavus* treated with different concentrations of PHE (Control, 8, 12, 16 mg/mL) for 24 h.

SEM was conducted to assess the effect of PHE on *A. flavus* cell morphology. As shown in Figure 3, the surface of *A. flavus* mycelium in the control group was dense, thick, and intact. In contrast, the surface of the mycelium was rough and had grooves after treatment with PHE. Specifically, at a concentration of 16 mg/mL, the mycelium surface displayed roughness, dryness, and folds. These results indicated that PHE caused significant damage to the cell structure of *A. flavus* mycelium. The mechanism of antifungal action may primarily involve cytoplasmic contraction and alteration of cell membrane structure, ultimately leading to cell death. As the concentration of PHE increased, *A. flavus* exhibited progressively more pronounced morphological abnormalities. PHE exerted significant disruptive effects on cellular integrity, resulting in the leakage of intracellular contents. Similarly, *Bacillus velezensis* E2, a lipopeptide-producing strain, demonstrated significant anti-aflatoxin activity by inhibiting *A. flavus* spore germination, inducing abnormal hyphal swelling, and causing cellular rupture [35]. Yang et al. have also confirmed by SEM that *Saccharomyces cerevisiae* NJ-1 VOC-treated *A. flavus* showed severe abnormalities in mycelium morphology, including uneven surface, irregular thickness, and complete inhibition of spore formation [36].

### 3.5. Effect of PHE on the Cell Membrane Integrity of A. flavus

**Figure 4:**
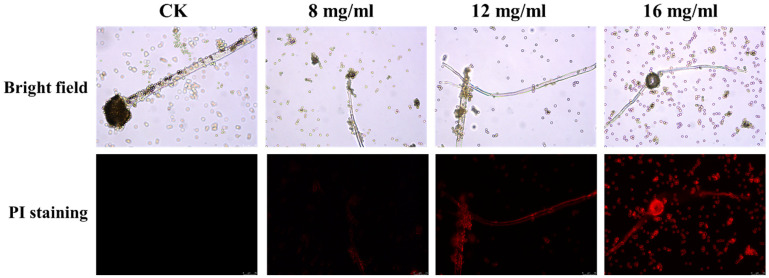
Effect of PHE on cell membrane of *A. flavus*. Cell membrane integrity of *A. flavus* analyzed using PI staining after 12 h of incubation with different concentrations of PHE.

To investigate the antifungal mechanism of PHE against *A. flavus*, propidium iodide (PI) staining was employed to assess cell membrane integrity (Figure 4). The results revealed an absence of red fluorescence in the control group, while the PHE-treated mycelia exhibited pronounced red fluorescence. As the concentration of PHE increased, the intensity of red fluorescence became more pronounced. This finding indicates that PHE treatment induced damage to the cellular membrane, consequently causing leakage of nucleic acid and protein in *A. flavus*, suggesting a potential mechanism of action involving impairment of membrane integrity.

### 3.6. Effect of PHE on the Maize Seed Infection by A. flavus

**Figure 5:**
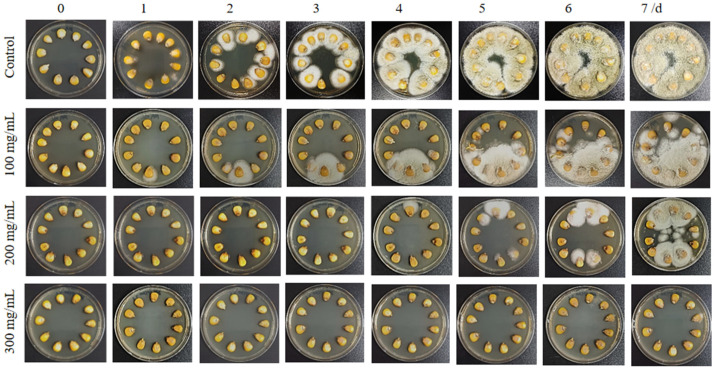
Comparison of different concentrations of PHE on maize seeds after infection with *A. flavus* contamination.

The effect of PHE on *A. flavus* contamination of maize seeds was then investigated. Changes in the morphology of *A. flavus* were observed after incubation for 7 days at different concentrations of PHE. The results demonstrated that PHE treatment significantly reduced aflatoxin contamination on maize seeds (Figure 5). Different doses of PHE (100, 200, and 300 mg/mL) inhibited the growth of *A. flavus* differently compared with the control values. Notably, the growth of *A. flavus* was completely inhibited in maize seeds treated with PHE (300 mg/mL) as compared to the control. There was no visible growth of *A. flavus* on maize seeds, while the control group treatment showed visible white mycelium on maize seeds. Furthermore, in the other treatment groups, even when a single maize seed was infected, infection did not spread rapidly throughout the plate. This suggested that the treatments were effective in limiting the spread of infection. Harvested maize is susceptible to pathogenic microorganisms. In particular, contamination by fungi such as *A. flavus* can produce the toxic and harmful substance aflatoxin [37]. Therefore, these findings concluded that the antifungal metabolite produced by *Pseudomonas* sp. HP-1 can be effectively applied as a partially purified extract (PHE), significantly reducing the cost associated with the purification process. This finding suggests that PHE is able to inhibit the antifungal compounds of *Aspergillus* sp. in a much simpler process. The results were in good agreement with the experiments carried out by Sawei, in which treatment of maize seeds with *Streptomyces* sp. culture filtrates reduced the development of fungi in stored maize seeds. [17]. PHE has strong inhibition of *A. flavus*, which may provide a new strategy for the prevention and control of maize diseases.

### 3.7. Isolation and Structural Determination of Antifungal Compound in the PHE

**Figure 6:**
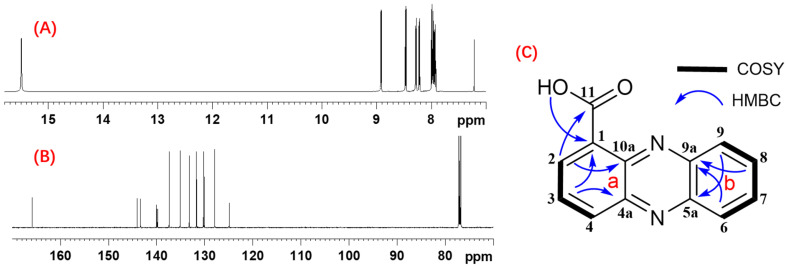
NMR data for PHE A. (**A**) ^1^H NMR spectrum of PHE A. (**B**) ^13^C NMR spectrum of PHE A. (**C**) COSY and key HMBC correlations for PHE A.

The PHE exhibited significant antifungal activity against *A. flavus*, demonstrating a pronounced absorption peak at a retention time of 13.6 min during HPLC analysis. The extracts underwent sequential fractionation via silica gel chromatography, yielding seven fractions. Notably, the second fraction exhibited antifungal properties against *A. flavus* and matched the high absorption peak at the same retention time in HPLC. Therefore, the crude fraction was purified by preparative HPLC. Finally, the pure compound, designated as PHE A, was elucidated and characterized using mass spectrometry, as well as one- and two-dimensional NMR spectroscopy. The compound of PHE A was obtained as a yellow solid. The molecular formula was determined as C_13_H_8_N_2_O_2_ on the basis of the HRESITOFMS analysis, which gave a sodium adduct ion [M + Na]^+^ at *m*/*z* 247.0477 (calculated for C_13_H_8_N_2_O_2_Na, 247.0483). The combined analysis of ^1^H (Figure S3) and ^13^C (Figure S4) NMR and HSQC spectral data revealed the presence of one exchangeable proton (δ_H_ 15.53) and seven aromatic methine groups (δ_H_ 7.90, 7.94, 7.96, 8.19, 8.26, 8.44, 8.89). The ^13^C NMR spectrum displayed resonances for one carbonyl carbon (δ_C_ 165.9), seven non-protonated sp^2^ carbons (δ_C_ 144.0, 143.3, 139.9, 139.8, 124.9), and seven sp^2^ methine carbons (δ_C_ 137.4, 135.1, 133.2, 131.7, 130.2, 130.0, 127.9). The a-ring was established by COSY correlations among H2/H3/H4, showing a doublet-triplet-doublet coupling typical for 1-, 2-, and 3-trisubstituted benzene protons, together with HMBC correlations from H2 to C10a, H3 to C1 and C4a. Further HMBC correlations from H2 to carbonyl carbon C11 and exchangeable proton 11-OH to C1 suggested that a carboxylic acid group should be attached to C1. The b-ring was assembled from COSY correlations H6/H7/H8/H9 and HMBC correlations from H6 and H8 to C9a, and H9 to C5a. Finally, the structure of PHE A was determined to be phenazine-1-carboxylic acid (PCA) (Figure 6) [38] on the basis of almost identical ^13^C chemical shift data, as shown in Table S1.

Phenazines represent a broad class of nitrogen-based heterocyclic secondary metabolites exhibiting redox-active aromatic properties, which are produced by diverse bacterial species through specialized biosynthetic pathways. Owing to their broad spectrum of biological activities, these compounds exhibit significant potential for applications in both agriculture and pharmaceuticals [39]. Among them, PCA has garnered considerable attention due to its high efficacy, low toxicity, and environmental compatibility, making it a promising agent for the prevention of fungal diseases across a wide range of crops [40]. The natural product PCA, an important microbial metabolite, is widely present in the secretions of microorganisms such as *Pseudomonads* and *Streptomycetes* [41]. The PCA has been demonstrated to exhibit remarkable structural stability and broad-spectrum antimicrobial activity against a variety of phytopathogens, including *Xanthomonas oryzae* (causing rice blight), *Acidovorax citrulli* (watermelon blight), *Phytophthora capsica* (chili pepper blight), *Fusarium graminearum* (wheat canker), *Colletotrichum orbiculare* (watermelon anthracnose), and *Mycosphaerella brassicicola* (rapeseed mycosphaerella) [42]. Furthermore, Gorantla et al. found that PCA exhibited excellent antifungal activity against *A flavus*, *C. albicans,* and *P. expansum* with zone of inhibition values of 17, 22, and 18 mm, respectively [43]. These properties underscore its potential as a versatile and effective agent for controlling plant diseases. Xun et al. have investigated the antifungal mechanism of PCA against *Pestalotiopsis kenyana* using transcriptomic analysis. Their findings revealed 3613 differentially expressed genes following PCA treatment, predominantly associated with redox processes and diverse metabolic pathways. Furthermore, PCA was observed to induce abnormal mycelial development, disrupt cell membrane integrity, reduce mitochondrial membrane potential, and elevate reactive oxygen species (ROS) levels [40]. In a separate study, Zhang et al. provided the first report on the marine-derived *Pseudomonas aeruginosa* PA31x, demonstrating its capability to synthesize PCA and regulate its biosynthesis via the *phz1* and *phz2* gene clusters [44]. These findings collectively underscore the multifaceted antimicrobial PCA.

## 4. Conclusions

In conclusion, the endophytic strain *Pseudomonas* sp. HP-1 isolated from *Peganum harmala* L. showed remarkable biocontrol capabilities. Its culture extract (PHE) exhibited potential antifungal properties and significantly inhibited the growth of fungal pathogens such as *P. italicum*, *A. niger*, and *A. flavus*. This highlights the potential of PHE as a sustainable biocontrol agent for the management of fungal diseases in agricultural applications. At a concentration of 16 mg/mL, PHE demonstrated near-complete inhibition of *A. flavus* mycelial growth. Furthermore, PHE significantly reduced the incidence of *A. flavus* contamination in stored agroecological maize seeds, with the treated PHE exhibiting enhanced antifungal activity compared to the untreated control, underscoring its potential as an effective antifungal agent for postharvest preservation. Through comprehensive NMR spectroscopy coupled with MS analysis, the primary bioactive constituent of PHE was identified as 1-phenazinecarboxylic acid (PCA), a compound known for its antimicrobial properties. This study provides a theoretical basis for the antifungal mechanism of PHE and highlights its potential application as a preservative in maize seed preservation.

## Data Availability

The original contributions presented in this study are included in the article/Supplementary Materials. Further inquiries can be directed to the corresponding author.

## Supplementary Materials

The following supporting information can be downloaded at: https://www.mdpi.com/article/10.3390/foods14101774/s1, Figure S1: HPLC-UV data for PHE; Figure S2: HRESIMS spectrum of PHE A (**1**); Figure S3: ^1^H NMR spectrum of **1** (600 MHz, CDCl_3_); Figure S4: ^13^C NMR spectrum of **1** (151 MHz, CDCl_3_); Figure S5: COSY spectrum of **1** (600 MHz, CDCl_3_); Figure S6: HSQC spectrum of **1** (600 MHz, CDCl_3_); Figure S7: HMBC spectrum of **1** (600 MHz, CDCl_3_); Table S1: Comparison of ^13^C chemical shifts of known PCA.
