# Supplementary material for: The Antimicrobial Extract Derived from Pseudomonas sp. HP-1 for Inhibition of Aspergillus flavus Growth and Prolongation of Maize Seed Storage
Source: Foods. 2025 May 16;14(10):1774. doi: 10.3390/foods14101774 (PMC12111000; doi:10.3390/foods14101774)
Supplement: Supplementary file 1 [file foods-14-01774-s001.zip › foods-3578972-supplementary.pdf]

Supplementary Information for

The antimicrobial extract derived from *Pseudomonas* sp. HP-1 for inhibition of *Aspergillus flavus* growth and prolongation of maize seed storage

Table of Contents

Figure S1. HPLC-UV data for PHE.
Figure S2. HRESIMS spectrum of PHE A (**1**).
Figure S3. ^{1}H NMR spectrum of **1** (600 MHz, $CDCl_3$).
Figure S4. ^{13}C NMR spectrum of **1** (151 MHz, $CDCl_3$).
Figure S5. COSY spectrum of **1** (600 MHz, $CDCl_3$).
Figure S6. HSQC spectrum of **1** (600 MHz, $CDCl_3$).
Figure S7. HMBC spectrum of **1** (600 MHz, $CDCl_3$).
Table S1. Comparison of ^{13}C chemical shifts of known PCA.

Figure S1. HPLC-UV data for PHE.

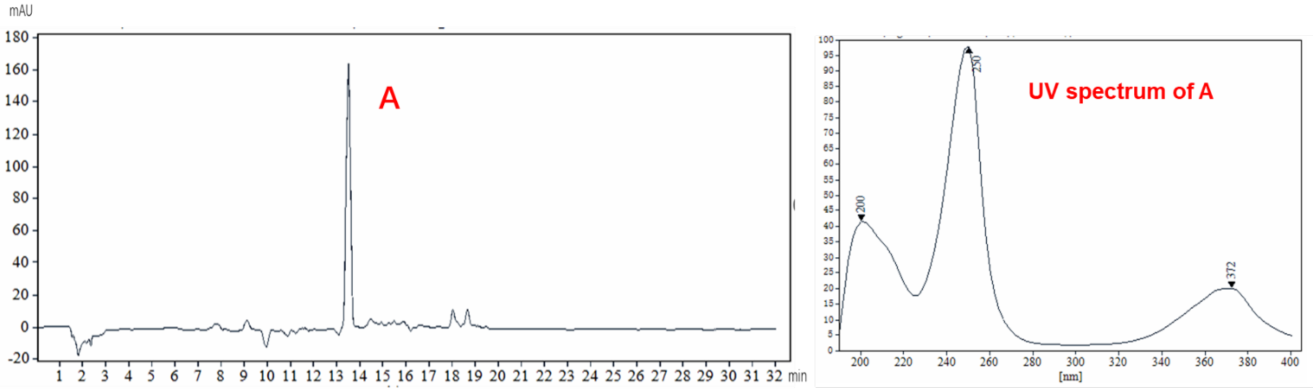

Figure S2. HRESIMS spectrum of PHE A (**1**).

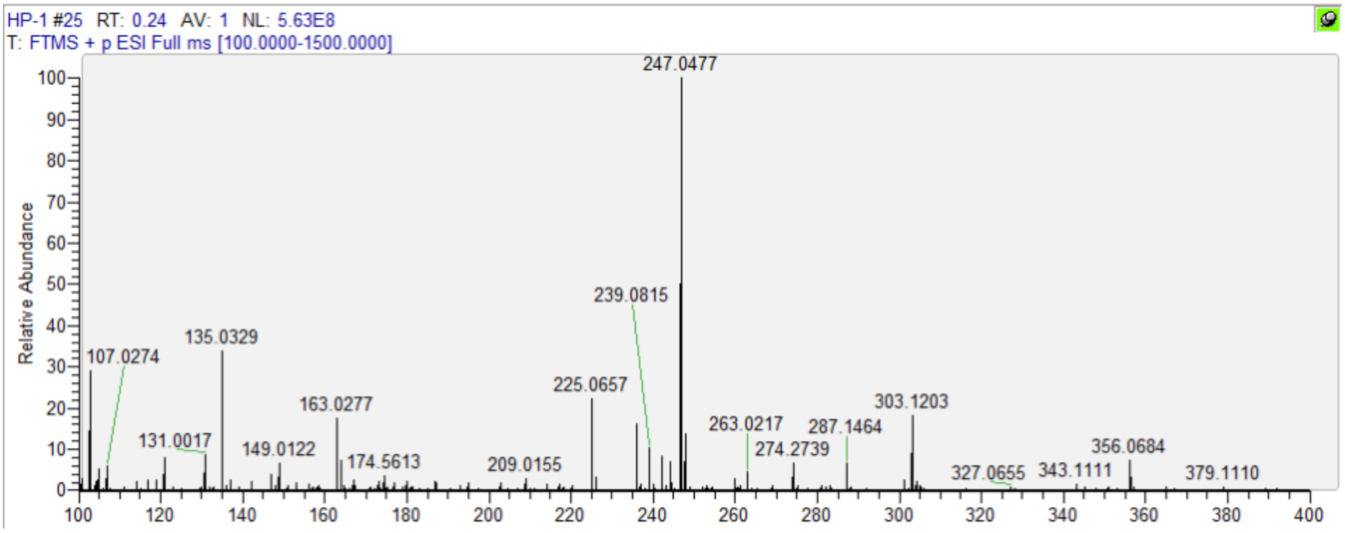

Figure S3. ^{1}H NMR spectrum of **1** (600 MHz, $CDCl_3$).

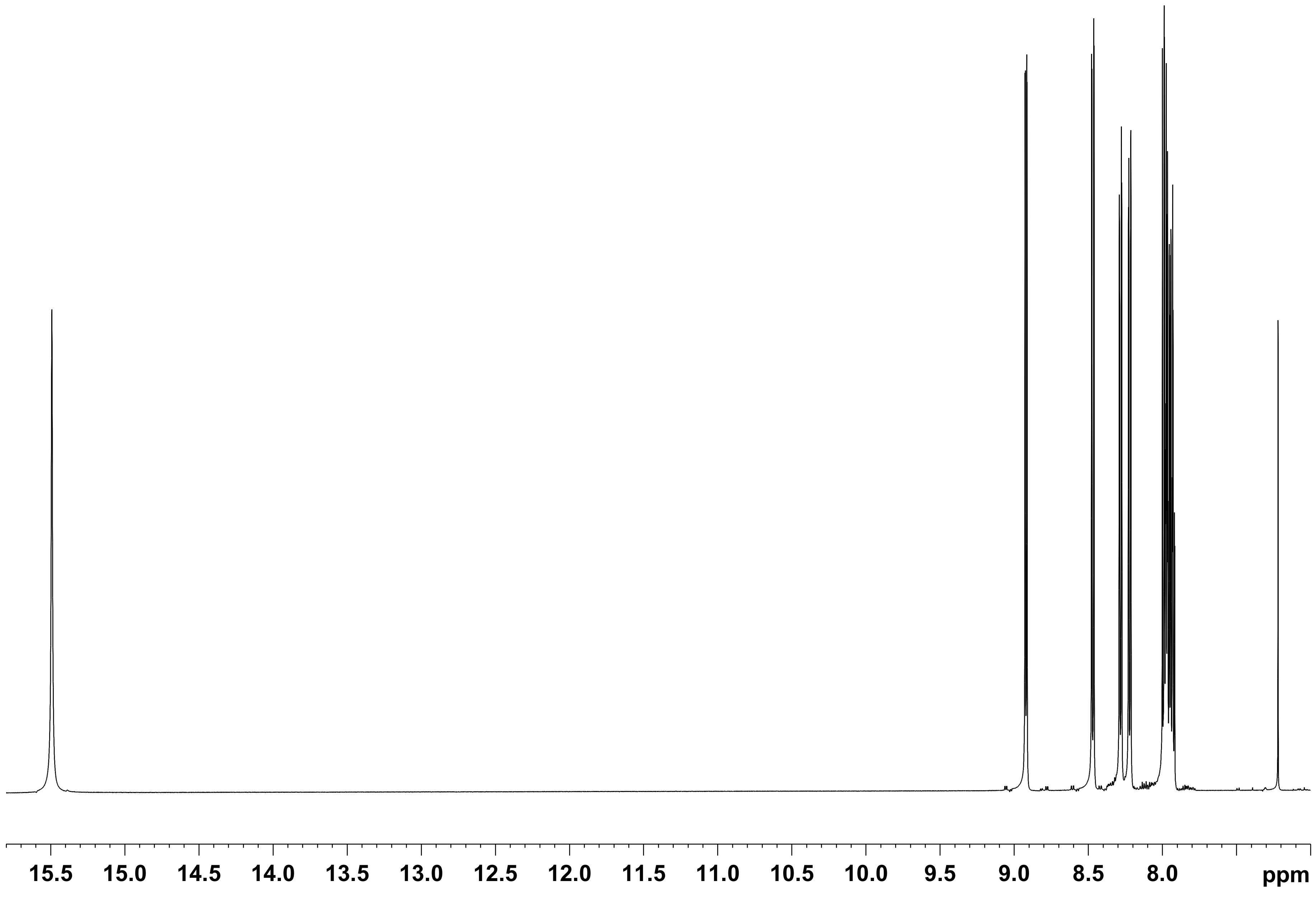

Figure S4. ^{13}C NMR spectrum of **1** (151 MHz, $CDCl_3$).

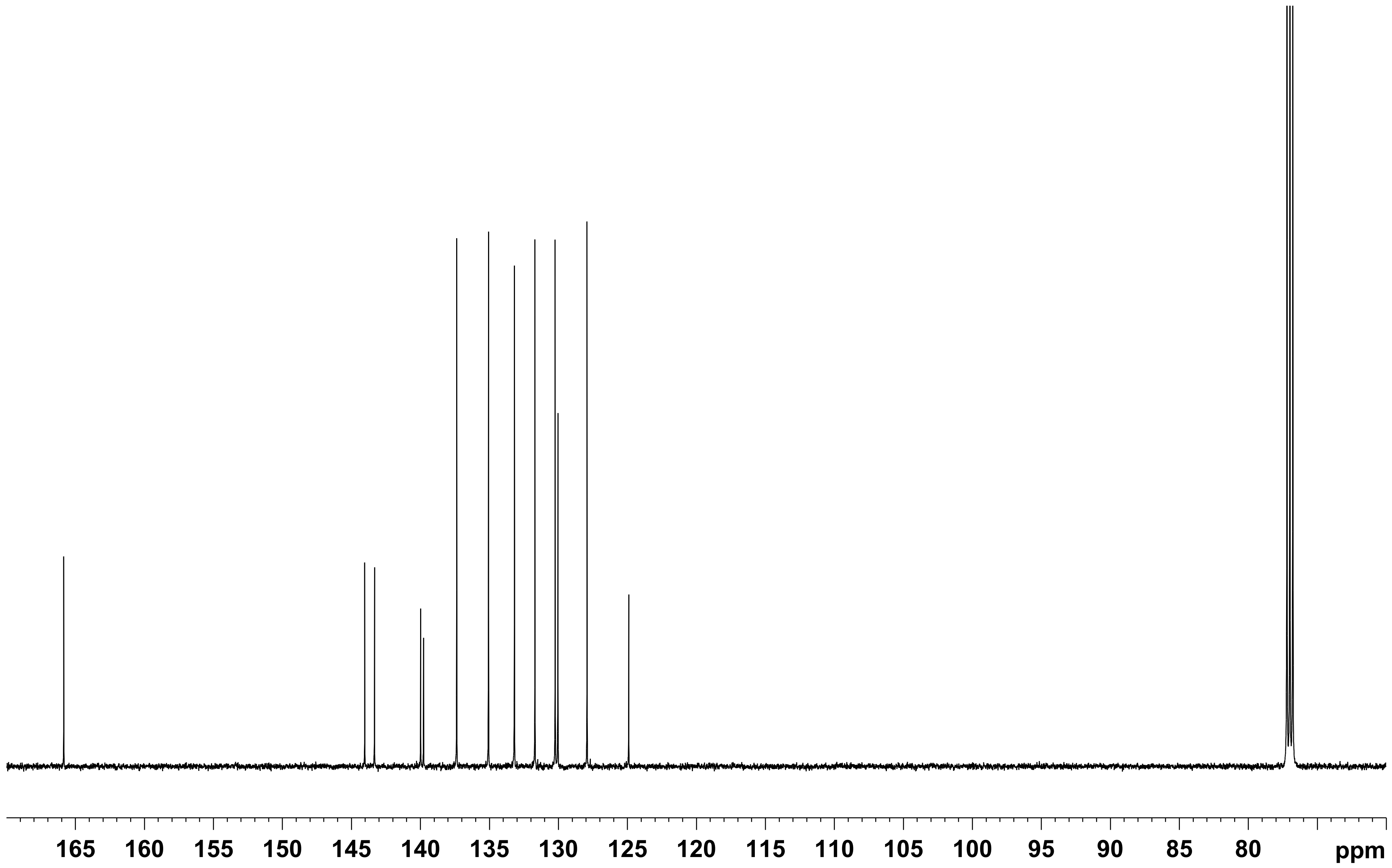

Figure S5. COSY spectrum of **1** (600 MHz, $CDCl_3$).

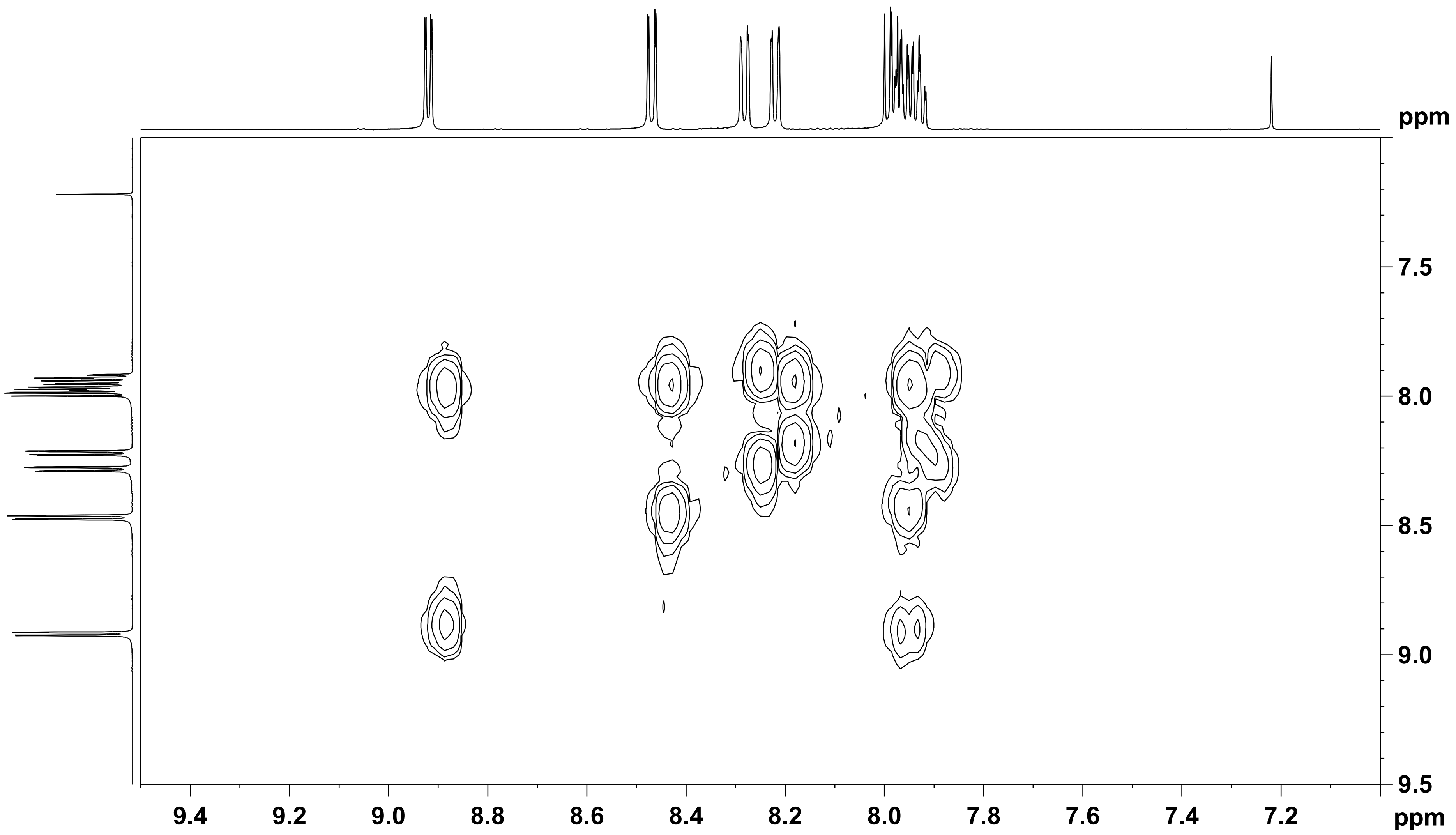

Figure S6. HSQC spectrum of **1** (600 MHz, $CDCl_3$).

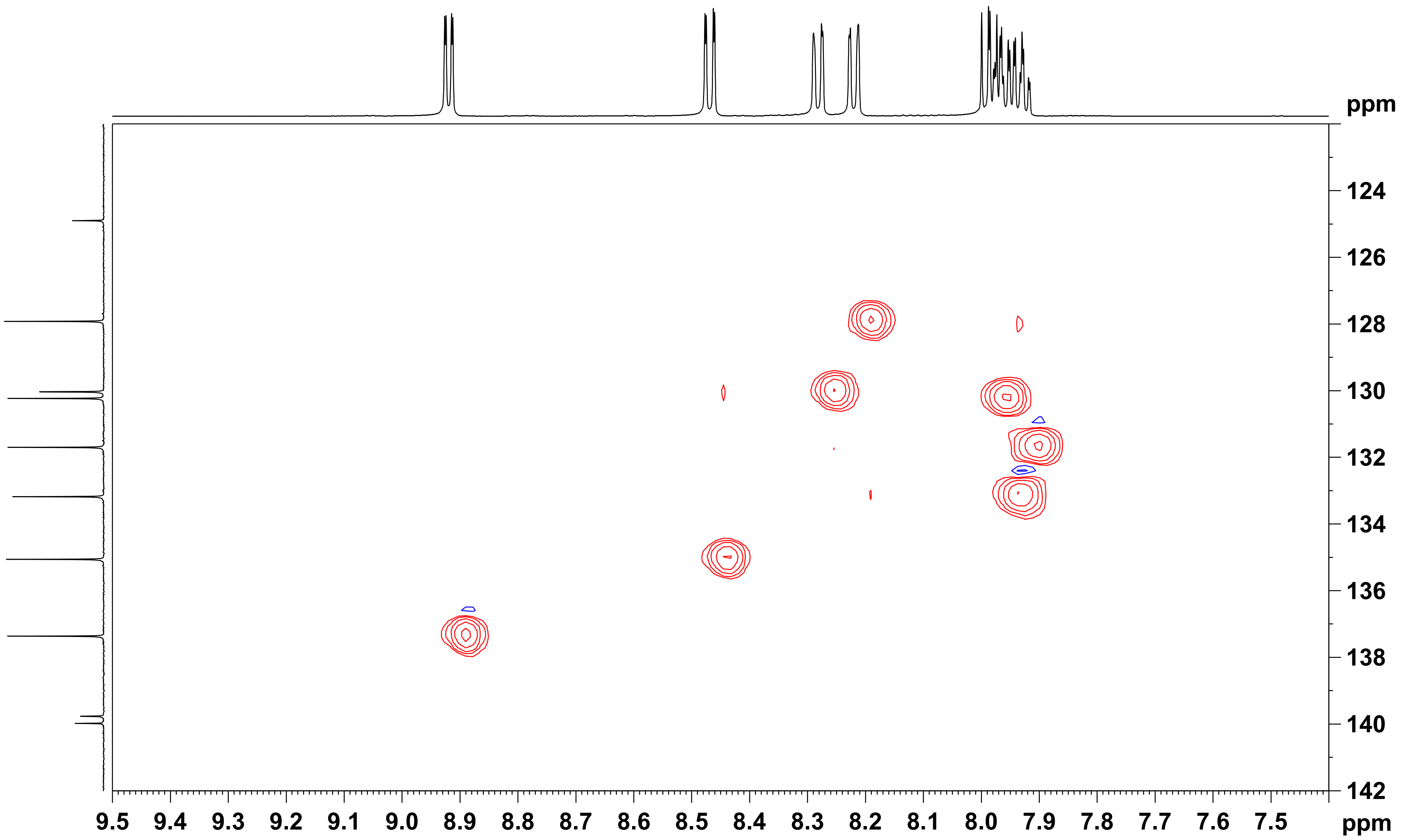

Figure S7. HMBC spectrum of **1** (600 MHz, $CDCl_3$).

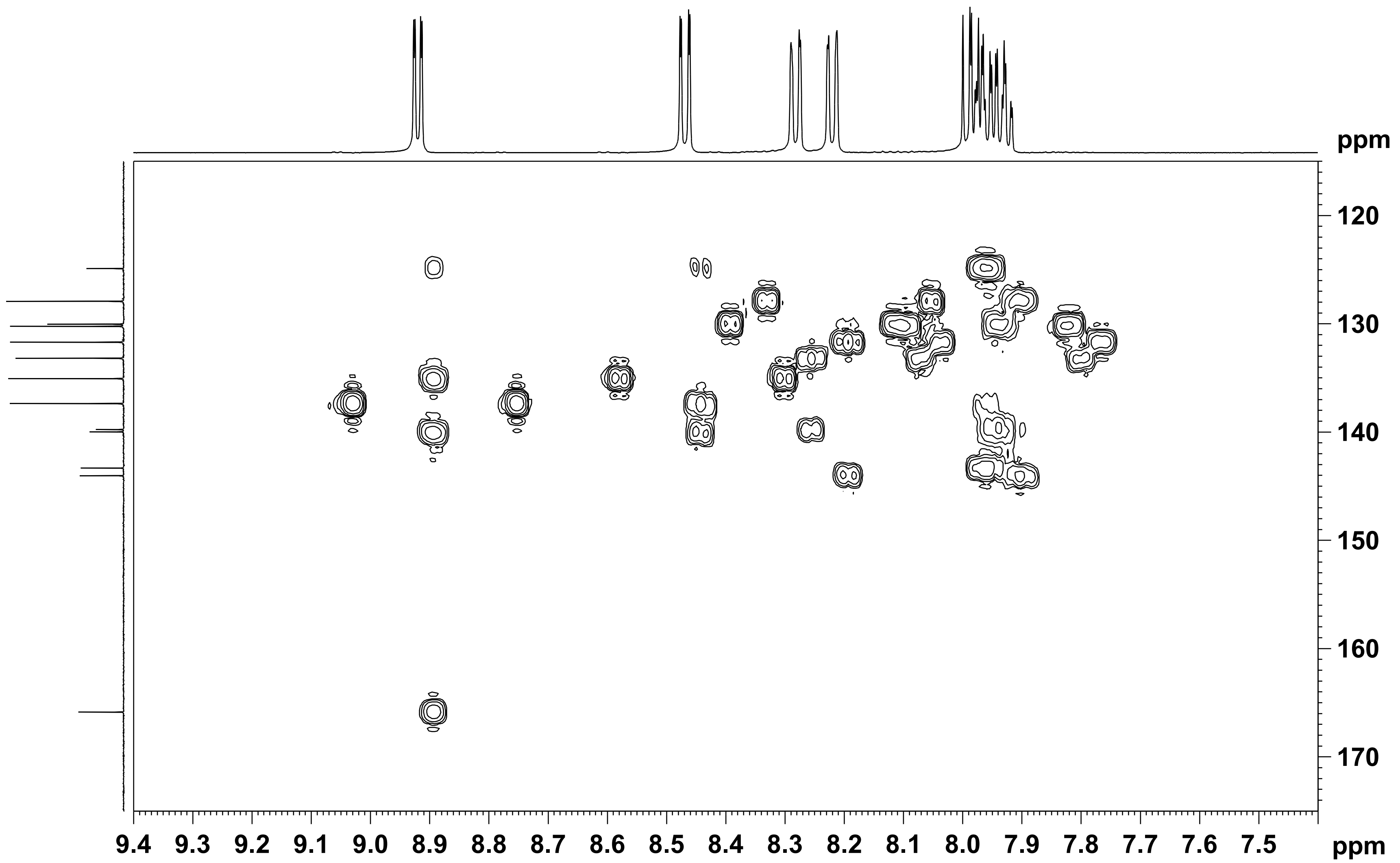

Table S1. Comparison of ^{13}C chemical shifts of PHE A (**1**) and known phenazine-1-carboxylic acid (PCA).

	PHE A (1)	PCA
no.	$\delta_C{}^b$	$\delta_C{}^b$
1	124.9	125.2
2	130.2	130.5
3	137.4	137.6
4	135.1	135.3
4a	139.7	140.1
5a	139.9	140.3
6	131.7	131.9
7	127.9	128.2
8	130.1	130.3
9	133.1	133.4
9a	143.3	143.6
10a	144.0	144.3
11	166.1	166.1
